# Pulmonary edema and blood volume after aneurysmal subarachnoid hemorrhage: a prospective observational study

**DOI:** 10.1186/cc8930

**Published:** 2010-03-23

**Authors:** Reinier G Hoff, Gabriel JE Rinkel, Bon H Verweij, Ale Algra, Cor J Kalkman

**Affiliations:** 1 Department of Perioperative & Emergency Care, Rudolf Magnus Institute of Neuroscience, University Medical Center Utrecht, Heidelberglaan 100, 3584 CX, Utrecht, The Netherlands; 2 Department of Neurology, Rudolf Magnus Institute of Neuroscience, University Medical Center Utrecht, Heidelberglaan 100, 3584 CX, Utrecht, The Netherlands; 3 Department of Neurosurgery, Rudolf Magnus Institute of Neuroscience, University Medical Center Utrecht, Heidelberglaan 100, 3584 CX, Utrecht, The Netherlands; 4 Julius Center for Health Sciences and Primary Care, University Medical Center Utrecht, Heidelberglaan 100, 3584 CX, Utrecht, The Netherlands

## Abstract

**Introduction:**

Pulmonary edema (PED) is a severe complication after aneurysmal subarachnoid hemorrhage (SAH). PED is often treated with diuretics and a reduction in fluid intake, but this may cause intravascular volume depletion, which is associated with secondary ischemia after SAH. We prospectively studied intravascular volume in SAH patients with and without PED.

**Methods:**

Circulating blood volume (CBV) was determined daily during the first 10 days after SAH by means of pulse dye densitometry. CBV of 60-80 ml/kg was considered normal. PED was diagnosed from clinical signs and characteristic bilateral pulmonary infiltrates on the chest radiograph. We compared CBV, cardiac index, and fluid balance between patients with and without PED with weighted linear regression, taking into account only measurements from the first day after SAH through to the day on which PED was diagnosed. Differences were adjusted for age, bodyweight, and clinical condition.

**Results:**

In total, 102 patients were included, 17 of whom developed PED after a mean of 4 days after SAH. Patients developing PED had lower mean CBV (56.6 ml/kg) than did those without PED (66.8 ml/kg). The mean difference in CBV was -11.3 ml/kg (95% CI, -16.5 to -6.1); adjusted mean difference, -8.0 ml/kg (95% CI, -14.0 to -2.0). After adjusting, no differences were found in cardiac index or fluid balance between patients with and without PED.

**Conclusions:**

SAH patients developing pulmonary edema have a lower blood volume than do those without PED and are hypovolemic. Measures taken to counteract pulmonary edema must be balanced against the risk of worsening hypovolemia.

**Trial registration:**

NTR1255.

## Introduction

Pulmonary edema (PED) is a severe complication in patients with a subarachnoid hemorrhage (SAH) from rupture of an intracranial aneurysm [1,2]. PED can result in severe hypoxemia and thus contribute to cerebral hypoxia in a brain that is already vulnerable to secondary injury. PED thereby increases the risk of poor outcome [2,3]. Next to such well-known causes of PED as cardiac failure or inflammatory reactions in the pulmonary tissue (for example, in sepsis), PED after SAH can have a neurogenic origin. Neurogenic PED is defined as an increase in interstitial and alveolar lung fluid occurring as a direct consequence of an acute central nervous system injury.

In the pathophysiology of neurogenic PED, several mechanisms are involved [1,4]. An abrupt increase in intracranial pressure or a localized ischemic insult in so-called neurogenic PED trigger zones, in the hypothalamus and medulla oblongata, leads to a massive sympathetic discharge. Severe systemic and pulmonary vasoconstriction ensues, with systemic hypertension and a marked increase in pulmonary hydrostatic pressure. This is followed by a fluid shift from the pulmonary capillaries into the lung tissue. Furthermore, the cerebral insult leads to inflammatory responses in the brain, with an increase in the production of brain cytokines, which can trigger inflammatory processes outside the brain. A systemic inflammatory response is accompanied by capillary leakage and the formation of generalized and pulmonary edema. The supraphysiologic sympathetic stimulation may also provoke cardiac dysfunctions with rhythm and conduction disturbances and mechanical pump failure, which contribute to the formation of PED.

Management of PED after SAH is centered on the traditional treatment strategies for cardiac failure-induced pulmonary edema, such as a reduction in preload and afterload and the use of inotropics [1,4,5]. A reduction in preload, by administration of diuretics and by a reduction in fluid intake, carries a risk of intravascular volume depletion. Patients after SAH already have a high risk of hypovolemia, and hypovolemia is associated with delayed cerebral ischemia and with poor outcome [6,7]. The avoidance of hypovolemia by ample fluid intake and, in some cases, the induction of hypervolemia, is therefore a mainstay of treatment after SAH [8]. To guide fluid management adequately, an accurate knowledge of volume status is required.

We assessed intravascular volume in patients after SAH and compared it between patients in whom PED did or did not develop.

## Materials and methods

### Study design and setting

We performed a prospective observational study in the UMC Utrecht, as a substudy of the Optica study. In that prospective controlled study, fluid management guided by daily measurements of circulating blood volume was compared with a fluid policy based on regular evaluations of the fluid balance, to assess its effect on the incidence of hypovolemia [9].

The study period was days 1 through 10 after the onset of SAH. The Medical Ethics Review Committee of the UMC Utrecht approved the study, and written informed consent was obtained.

### Study population

Patients were eligible if admitted to the UMC Utrecht within 72 h after aneurysmal SAH. Patients with accompanying head injury, pregnancy, liver or kidney failure, an allergy for the indicator dye indocyanine green, or with imminent death on admission were excluded. Patient data on demographic and clinical variables were collected prospectively. The clinical condition on admission was classified according to the World Federation of Neurological Surgeons scale [10]. Delayed cerebral ischemia (DCI) was defined as a decrease in level of consciousness of ≥ 2 points on the Glasgow Coma Scale, the appearance of a focal neurologic deficit, or both, for ≥ 3 hours, after exclusion of rebleeding, hydrocephalus, infection, or metabolic causes for the deterioration [11]. Neurologic outcome was assessed with the modified Rankin scale at 3 months after the SAH by a research nurse not involved in patient management [12].

### Treatment

Patients were treated according to a standard SAH protocol. The goal of fluid therapy was to maintain normovolemia. The guidance of fluid therapy depended on treatment allocation for the parent Optica study. In the control group, fluid administration was adjusted on the basis of the fluid balance, calculated every 6 hours, by subtracting urinary volume from total oral and intravenous intake. The aim was to keep the daily fluid balance at 750 ml positive, to compensate for insensible fluid loss. In the intervention group, fluid management was adjusted on the basis of daily blood-volume measurements, in an effort to keep blood volume inside the normovolemic range. Central venous pressure was not routinely monitored in patients with good or reasonable neurologic condition (WFNS grades I and II). Oral nimodipine, 60 mg every 4 hours, was started in all patients. If signs of delayed cerebral ischemia developed, fluid management was continued according to treatment allocation; no intentional hypervolemia or hemodilution was used, but induced hypertension could be applied. Treating physicians could overrule the protocol-based fluid therapy in both study groups if they considered this vital to the patient's interest.

### Outcomes

A diagnosis of PED was made by the treating physicians and the consulting radiologists, based on clinical signs (dyspnea, tachypnea, basal pulmonary crackles, presence of frothy sputum, hypoxemia) in combination with characteristic bilateral pulmonary infiltrates on the chest radiograph [4]. Presence of pneumonia excluded a diagnosis of PED.

Blood volume and cardiac output were measured daily in all included patients by means of pulse dye densitometry (PDD; Nihon Kohden Corporation, Tokyo, Japan). This dye-dilution technique uses pulse spectrophotometry, as developed for pulse oximetry, for measurement of the concentration of an injected dye (indocyanine green; SERB Laboratoires Pharmaceutiques, Paris, France). PDD was previously validated, showing good accuracy in measured blood volume, and was used before in patients after SAH [7,13]. A measured blood volume of 60 to 80 ml/kg was classified as normal, as was a cardiac index of 2.5 to 4 L/min/m^2 ^[14].

### Data analysis

We calculated individual per-patient mean-blood-volume values for all patients developing pulmonary edema, taken into consideration only the blood-volume measurements from the first day after SAH through the day on which pulmonary edema was diagnosed for the first time in that patient. We calculated the median number of days after SAH after which pulmonary edema was diagnosed. We then assessed the per-patient mean-blood-volume values for patients in whom pulmonary edema did not develop, based on the measurements made until this median number of days after SAH. To compare mean values between patients with and without pulmonary edema, we took into account that, in each patient, multiple blood-volume measurements were performed during the study period. Therefore we used weighted linear regression for comparison of per-patient mean values, in which the inverse of the standard error of the per-patient mean was taken as weight.

Advanced age, obesity, and a poor clinical grade on admission have all been associated with a decrease in blood volume. As we aimed to analyze the relation between PED and volume status, we decided to adjust for these variables (age, weight, WFNS-grade). A similar analysis was used for comparison of cardiac index and fluid balance in all patients, to compare measurements of patients with pulmonary edema between intervention and control groups of the Optica study, and to compare measurements in patients in whom PED developed on the first day after SAH or on later days. Results are presented as mean differences with corresponding 95% confidence intervals.

We compared the proportion of patients with pulmonary edema between the intervention and control groups in terms of risk ratio (RR), with 95% confidence intervals. The same analysis was used to compare the use of diuretics in patients in whom PED did or did not develop in the following days.

## Results

### Patient enrollment

**Figure 1:**
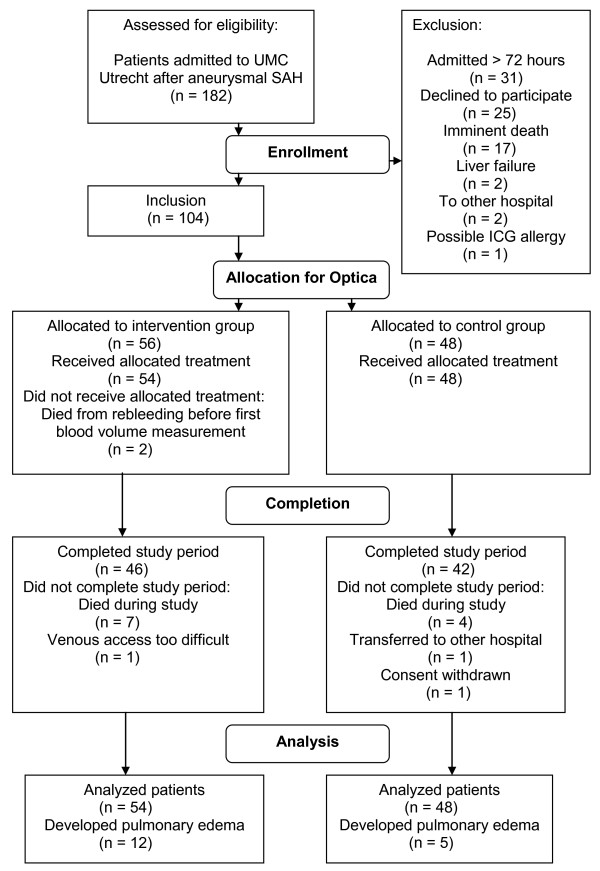
**Flow chart of patient inclusion and treatment allocation**.

Between January 2006 and June 2007, 182 patients with aneurysmal SAH were admitted to the UMC Utrecht. Of these, 104 patients were included, two of whom died of rebleeding before the first blood volume measurement (Figure 1). Baseline characteristics of patients with or without PED are listed in Table 1. Patients in PED developed were older and more often were admitted in a poor clinical condition. No difference was found in baseline characteristics between patients from the intervention and the control groups.

**Table 1:** Patient characteristics

	Pulmonary edema	No pulmonary edema
Number of patients	17	85
Women	14 (82%)	64 (75%)
Age (years, mean ± SD)	66 ± 14	55 ± 14
Length (cm, mean ± SD)	170 ± 8	171 ± 10
Weight (kg, mean ± SD)	79 ± 13	75 ± 17
		
Aneurysm location		
Anterior cerebral artery	7 (41%)	38 (45%)
Carotid artery	6 (35%)	20 (24%)
Middle cerebral artery	2 (12%)	15 (18%)
Posterior circulation	2 (12%)	12 (14%)
		
WFNS grade on admission		
I	5 (29%)	42 (49%)
II	5 (29%)	13 (15%)
III	0 (0)	7 (8%)
IV	4 (24%)	14 (17%)
V	3 (18%)	9 (11%)
		
Aneurysm treatment		
Coiling	10 (59%)	50 (59%)
Clipping	3 (18%)	28 (33%)
		
Delayed cerebral ischemia	9 (53%)	27 (32%)
		
Modified Rankin Scale at 3 months		
1	3 (18%)	30 (35%)
2	4 (24%)	19 (22%)
3	3 (18%)	9 (11%)
4	2 (12%)	3 (4%)
5	2 (12%)	5 (6%)
Dead	3 (18%)	16 (19%)

Data are expressed as numbers with percentages or means with standard deviations.

SD, standard deviation; WFNS, World Federation of Neurological Surgeons.

### Outcome measurements

Neurogenic pulmonary edema was diagnosed after a mean of 4.4 days (95% CI, 3.0 to 5.9) after SAH. Calculated differences in blood volume, cardiac index, and fluid balance between patients with and without PED are presented in Table 2. The mean blood volume of patients with PED was in the hypovolemic range, whereas the mean blood volume of patients without PED was in the normal range. After adjusting for age, weight, and WFNS grade, blood volumes remained lower in patients with PED, whereas cardiac index and fluid balance did not differ statistically significantly between patients with or without PED. No difference was noted in mean CBV between patients with PED diagnosed early and late.

**Table 2:** Calculated differences in outcome measurements

	Pulmonary edema			
			
	Diagnosed n = 17	Not diagnosed n = 85	Mean difference	Adjusted mean difference
Blood volume	56.6	66.8	-11.3	-8.0
	(52.3 to 60.8)	(64.1 to 69.4)	(-16.5 to -6.1)	(-14.0 to -2.0)
Cardiac index	2.6	3.2	-0.5	-0.3
	(2.2 to 2.9)	(3.1 to 3.4)	(-0.9 to -0.1)	(-0.7 to 0.1)
Fluid balance	+1.6	+1.1	+0.2	+0.1
	(1.0 to 2.2)	(0.9 to 1.3)	(-0.2 to 0.7)	(-0.4 to 0.6)

Presented are mean values with 95% confidence interval of blood volume (ml/kg), cardiac index (L/min/m^2^), and fluid balance (L/day). Differences between patients with or without pulmonary edema are adjusted for age, weight, and WFNS grade on admission.

n = numbers of patients.

PED was diagnosed in 17 (17%) of the 102 evaluated patients. It occurred in 12 (22%) of the 54 patients in the intervention group and in five (10%) of the 48 patients in the control group (RR, 2.1; 95% CI, 0.8 to 5.6). A comparison in determinants between both groups is provided in Table 3. The mean blood volume of the patients with PED in the intervention group was slightly higher than that in the control group, but still in the hypovolemic range. Diuretics were used in seven (8%) of 85 patients without PED and in 11 (65%) of 17 patients with PED, in the days before PED was diagnosed (RR, 7.9; 95% CI, 3.6 to 17.3).

**Table 3:** Comparison between patients with pulmonary edema from intervention and control groups of the Optica study

	Intervention group n = 12	Control group n = 5	Mean difference	Adjusted mean difference
Blood volume	58.2	52.0	7.9	9.1
	(53.4 to 63.0)	(39.2 to 64.8)	(0.7 to 15.1)	(-0.9 to 19.1)
Cardiac index	2.5	2.9	-0.5	-0.2
	(2.1 to 2.8)	(1.4 to 4.4)	(-1.3 to 0.2)	(-1.1 to 0.7)
Fluid balance	+1.9	+0.7	+1.2	+1.2
	(1.4 to 2.5)	(-1.0 to 2.5)	(0.1 to 2.3)	(-0.2 to 2.6)

Presented are mean values with 95% confidence interval of blood volume (ml/kg), cardiac index (L/min/m^2^) and fluid balance (L/day). Differences between patients from intervention or control group are adjusted for age, weight, and WFNS grade on admission.

*n*, numbers of patients.

## Discussion

Our results show that PED after SAH is accompanied by a strong decrease in blood volume. Patients with PED had a mean blood volume in the hypovolemic range in the days preceding the diagnosis of PED. Patients in whom PED developed more often had diuretics prescribed by the treating physicians in the days before the diagnosis of PED was made. Cardiac index tended to be somewhat lower and fluid balance somewhat more positive in patients with PED, but these differences were no longer statistically significant after adjusting for age, weight, and clinical condition. Calculations were based on measurements obtained in the time period from the day after SAH until the day PED was diagnosed, so these results were not influenced by any therapeutic measures initiated by the treating physicians to counteract PED.

We were unable to find any previous studies in which blood volume was actually measured in PED patients, either in patients with a cardiac origin of the edema (by systolic or diastolic pump failure), or in patients with noncardiogenic edema (by increased pulmonary capillary permeability). Central venous pressure and pulmonary capillary occlusion pressure are often used in PED to inform about volume status [15]. However, no clear relation exists between these pressures and the presence of neurogenic PED, or between these pressures and measured blood volume [5,16,17].

It was previously noticed that neurogenic PED develops earlier in patients with a normal systemic circulating volume, compared with hypovolemic patients [1,18]. Patients in the Optica intervention group more often had blood volumes in the normovolemic range than did control group patients [9]. The present analysis showed that intervention-group patients tended to be at increased risk of PED and that patients in the intervention group developing PED tended to have a more positive fluid balance and a higher (but still reduced) mean blood volume. Interpretation of these differences must be made with caution because of the small number of patients in this comparison. However, this might indicate that measures to prevent hypovolemia after SAH lead to an increased risk of PED.

The cardiac index was slightly (nonsignificantly) lower in patients with PED than in those without, but not to such an extent as to indicate cardiac failure. This suggests that PED in our study did not have a cardiogenic origin, but was likely neurogenic or part of a systematic inflammatory response syndrome (SIRS). The reduced blood volume we found in patients with PED also suggests a noncardiogenic cause. A reduction in cardiac index could be explained by the cardiac dysfunction often accompanying neurogenic PED. Alternatively, preexisting hypovolemia may have led to a decrease in cardiac filling and thereby to a reduction in cardiac index. Because we did not perform serial echocardiography, we were unable to make this distinction.

The reduction in blood volume we observed in patients with PED after SAH does not necessary imply a causal relation. Patients in whom PED developed were older and more often admitted in a poor clinical condition, which is in agreement with previous studies [4]. Ill patients tend to develop more signs of SIRS, with capillary leakage and the formation of a generalized edema [19]. This loss of fluid from the circulation reduces blood volume. Fluid retention is the usual reaction of the body to a reduction in volume status, thereby leading to a more-positive fluid balance. Both the presence of generalized edema and a positive fluid balance could erroneously be interpreted as a sign of fluid overload in a patient with SIRS. This could trigger the treating physicians to deviate from the fluid-management protocol and to use diuretics. A previous study found no association between the fluid balance and actual measured blood volume [20]. In our present study, diuretics were used far more often in patients in whom, in later days, PED developed than in patients without edema. We did not collect information on the motives for the use of diuretics. However, the use of diuretics in patients with already impending hypovolemia may have played an important role in the reduction in intravascular volume that we observed.

Furthermore, any formation of PED implies a fluid shift from the vascular system to the lung tissue. Therefore, the occurrence of PED could contribute to a further reduction in blood volume.

A limitation of our study is that the diagnosis of PED was based on clinical criteria in combination with chest radiograph findings. The physicians responsible for patient care were not blinded for treatment allocation. This may have contributed to an increase of PED diagnosed in the intervention group, because some clinicians believed that patients in the intervention group were at increased risk for hypervolemia and pulmonary edema. We did not categorize the severity of edema. Less-severe instances of pulmonary edema may have escaped detection, as extravascular lung water must increase by >30% for edema to be visible on a chest radiograph [15]. We did not routinely monitor central venous pressure or pulmonary capillary wedge pressure in these patients, because of the poor relation between these pressures and actual volume status. Another limitation concerns the definition of normal blood volume. Previous studies found "normal" blood volume values in adults of ~70 ml/kg [7,14]. However, the changes in blood volume that occur as a result of illness or therapy are incompletely understood [21]. For this reason, we used wide margins in our definition of normovolemia (60-80 ml/kg). Even with these large margins, patients developing PED fell outside this range and were considered hypovolemic.

Notably, volume status is only one of the factors determining the adequacy of tissue perfusion. To evaluate the patient's cardiovascular status, many hemodynamic parameters must be taken into consideration together and seen in the context of the overall clinical condition.

## Conclusions

Patients with PED after SAH must be considered hypovolemic and therefore at increased risk of delayed cerebral ischemia. This renders fluid management in these patients especially difficult. Measures aimed at relieving pulmonary congestion and improving oxygenation (for example, preload reduction) might increase hypovolemia, whereas measures to improve volume status might worsen pulmonary edema and possible hypoxemia. Both hypovolemia and hypoxemia are extremely deleterious for the recently injured brain. To balance these potentially conflicting goals, clinicians might consider refraining from measures that reduce preload and instead use early (noninvasive) positive-pressure ventilation to maintain oxygenation. This might allow administration of additional fluid to maintain normovolemia without a further decrease in arterial oxygen saturation. The effects of such a treatment policy on circulation, ventilation, and neurologic outcome must be formally studied.

## Key messages

• Patients with pulmonary edema after aneurysmal SAH have a decrease in circulating blood volume.

• These patients must be considered at high risk of delayed cerebral ischemia.

• Treatment policies for pulmonary edema after SAH must be balanced against the risk of further increasing hypovolemia.

## Abbreviations

CBV: circulating blood volume; DCI: delayed cerebral ischemia; PDD: pulse dye densitometry; PED: pulmonary edema; SAH: subarachnoid hemorrhage; UMC: University Medical Center; WFNS: World Federation of Neurological Surgeons.

## Competing interests

The authors declare that they have no competing interests.

## Authors' contributions

All of the authors were involved in designing the study. RH collected the data and drafted the manuscript. AA was involved in statistical analysis. All authors were involved in interpretation of the data. GR, BV, AA, and CK revised the manuscript. All authors approved the final manuscript.

## Authors' information

RGH is an anesthesiologist-intensivist at the UMC Utrecht, the Netherlands. GJER is a professor in neurology at the UMC Utrecht, the Netherlands. BHV is a neurosurgeon at the UMC Utrecht, the Netherlands. CJK is a professor in anesthesiology at the UMC Utrecht, the Netherlands. AA is a professor in clinical epidemiology at the UMC Utrecht, the Netherlands.
